# The Sasaki-W anastomosis for recurrent Crohn’s disease stenosis after the Kono-S anastomosis

**DOI:** 10.1186/s40792-023-01747-z

**Published:** 2023-09-29

**Authors:** Takahiro Asai, Hiroharu Shinozaki, Satoshi Shinozaki, Akitsugu Makino, Masashi Nakagawa, Kenji Kobayashi, Alan Kawarai Lefor, Seigo Yukisawa, Yoshiro Ogata

**Affiliations:** 1 Department of Surgery, Saiseikai Utsunomiya Hospital, 911-1 Takebayashi, Utsunomiya, Tochigi 321-0974 Japan; 2 Shinozaki Medical Clinic, Utsunomiya, Japan; 3 Department of Surgery, Jichi Medical University, Shimotsuke, Japan; 4 Department of Medical Oncology, Saiseikai Utsunomiya Hospital, Utsunomiya, Japan

**Keywords:** Crohn’s disease, Kono-S anastomosis, Sasaki-W anastomosis, Resection, Stricturoplasty

## Abstract

**Background:**

Postoperative recurrence is frequently encountered in the management of patients with Crohn’s disease and is most often found at the anastomotic site. A novel technique, the Sasaki-W anastomosis, is an antimesenteric cutback end-to-end isoperistaltic anastomosis. We report a patient with Crohn’s disease who underwent partial intestinal resection for postoperative anastomotic stenosis, reconstructed with the Sasaki-W anastomosis, after initial intestinal resection reconstructed with a Kono-S anastomosis.

**Case presentation:**

A 30-year-old male was diagnosed with Crohn’s disease and treated with mesalamine and adalimumab, and he underwent ileocecal resection using the Kono-S anastomosis at the time of diagnosis. He was treated with infliximab without any symptoms or recurrence for 7 years. He was admitted presenting with upper abdominal pain. Physical examination showed mild tenderness and distension in the upper abdomen. Laboratory data showed no remarkable findings. Computed tomography scan showed wall thickening in the ileum with proximal dilation and fluid retention. Non-operative management with antibiotics and fasting did not improve the symptoms within 7 days. Ten days after admission, ileocecal resection reconstructed with the Sasaki-W anastomosis was performed. At operation, there was a 15-cm intestinal stenosis at the site of the previous Kono-S anastomosis. The transverse colon and ileum were reconstructed with the Sasaki-W anastomosis. The postoperative course was uneventful, and the patient was discharged 17 days postoperatively. The patient had no obstructive symptoms and no findings consistent with bowel obstruction were observed on computed tomography scan one year postoperatively.

**Conclusions:**

The Sasaki-W anastomosis is a viable option for intestinal reconstruction in patients with postoperative recurrence after a Kono-S anastomosis.

## Background

Crohn’s disease (CD) is a chronic inflammatory bowel disease that affects the entire gastrointestinal tract. A long-term study reported that 71% of patients with CD undergo surgery within 10 years after the initial diagnosis, and postoperative recurrence occurred in 44% after resection [1]. Postoperative recurrence is frequent in the long-term management of patients with CD and it is more likely to occur at the site of anastomosis [2]. Currently, there is no consensus about the best approach to prevent postsurgical recurrence of CD. Several variables have been identified as potential factors influencing surgical outcomes, including smoking habits, bowel perforation and perianal fistulas [3]. Among these factors, it is of interest to speculate whether the specific surgical techniques used could reduce the rate of recurrence or not. The Kono-S anastomosis, an antimesenteric functional hand-sewn end-to-end anastomosis, was introduced in 2011 and has attracted attention in recent years as it appears to have low complication and recurrence rates [4]. The Kono-S anastomosis has been performed and is popular around the world. In 2021, Sasaki et al. introduced a new antimesenteric cutback end-to-end isoperistaltic anastomotic technique known as the Sasaki-W anastomosis [5]. Here, we report a patient with CD who underwent a repeat partial intestinal resection reconstructed with a Sasaki-W anastomosis which was undertaken due to postoperative anastomotic stenosis after an initial resection reconstructed with the Kono-S anastomosis.

## Case presentation

**Fig. 1:**
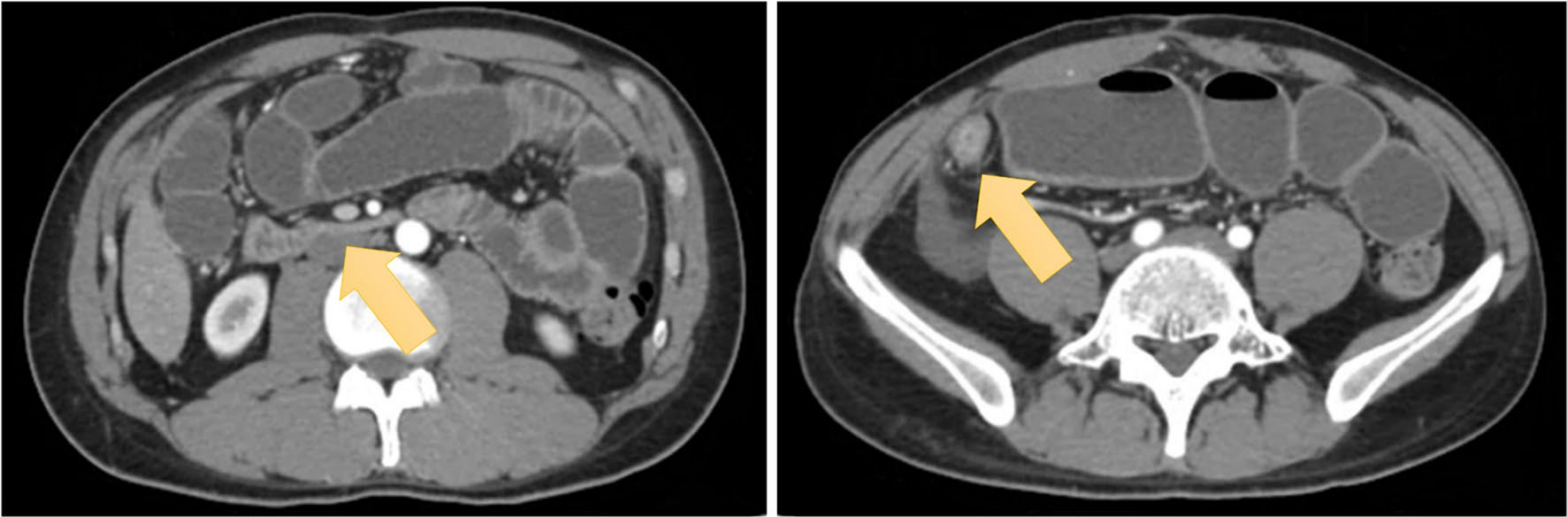
Contrast-enhanced abdominal computed tomography scan shows wall thickening in the ileum with signs of obstruction (arrows)

**Fig. 2:**
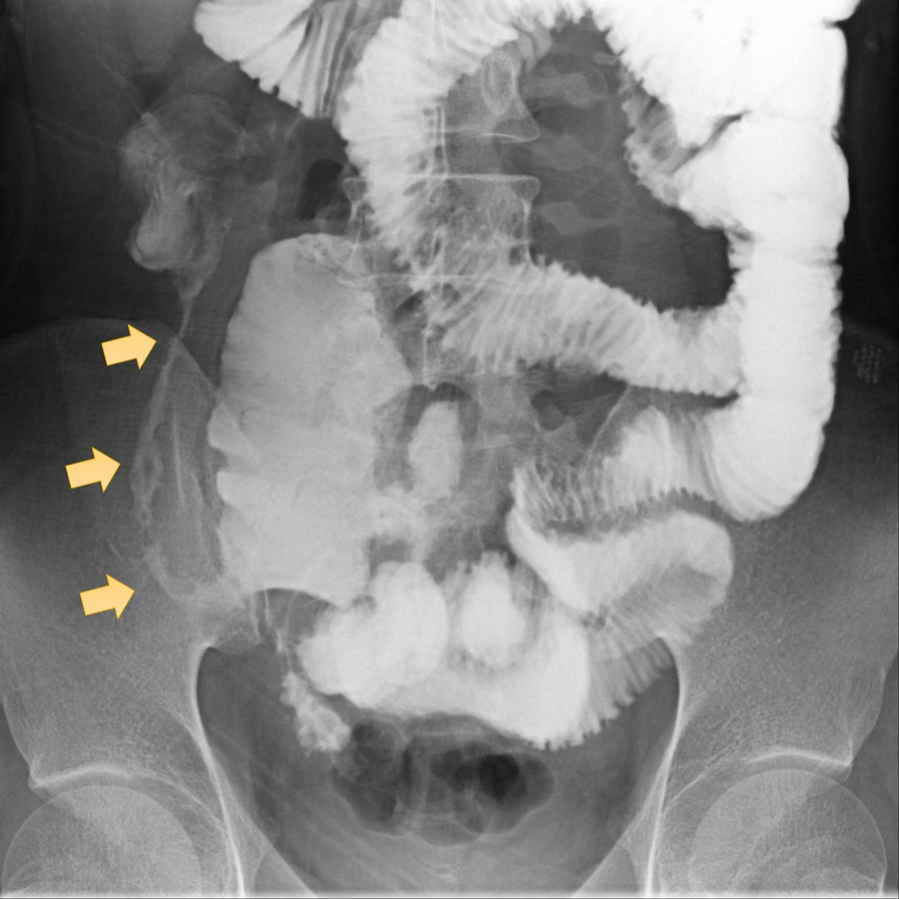
Gastrointestinal series shows stenosis of the distal ileum 7 days after admission

**Fig. 3:**
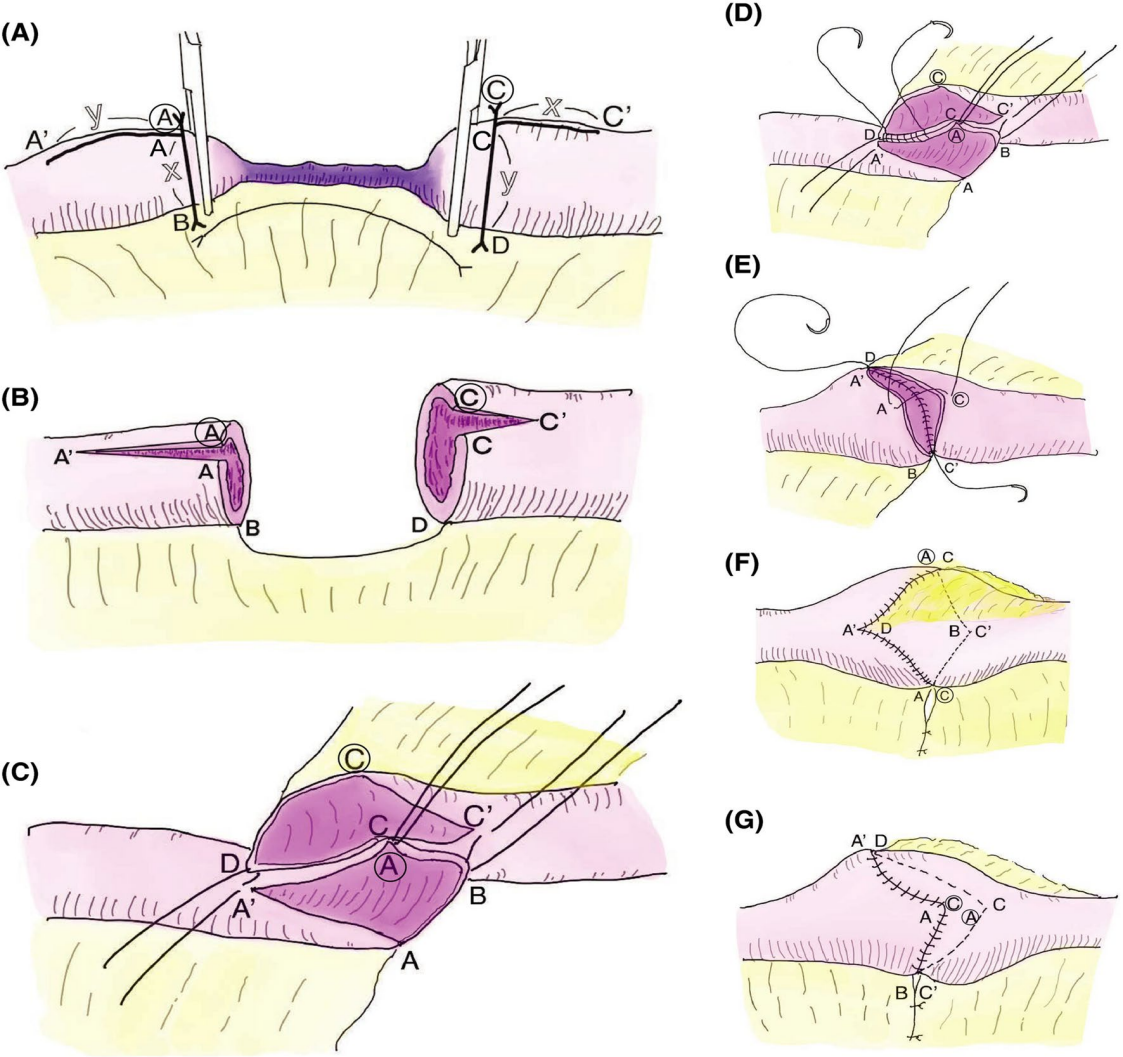
A Schema of procedure for the Sasaki-W anastomosis (Ref [5], with permission). **A** Measurement and marking of crushed bowel length. **B** Antimesenteric cutback-incision. **C** Supporting suture setup. **D** Suturing of the posterior wall with supporting suture. **E** Suturing of the anterior wall with supporting suture. **F** Completion of the anastomosis (upper view). **G** Completion of the anastomosis (lateral view)

**Fig. 4:**
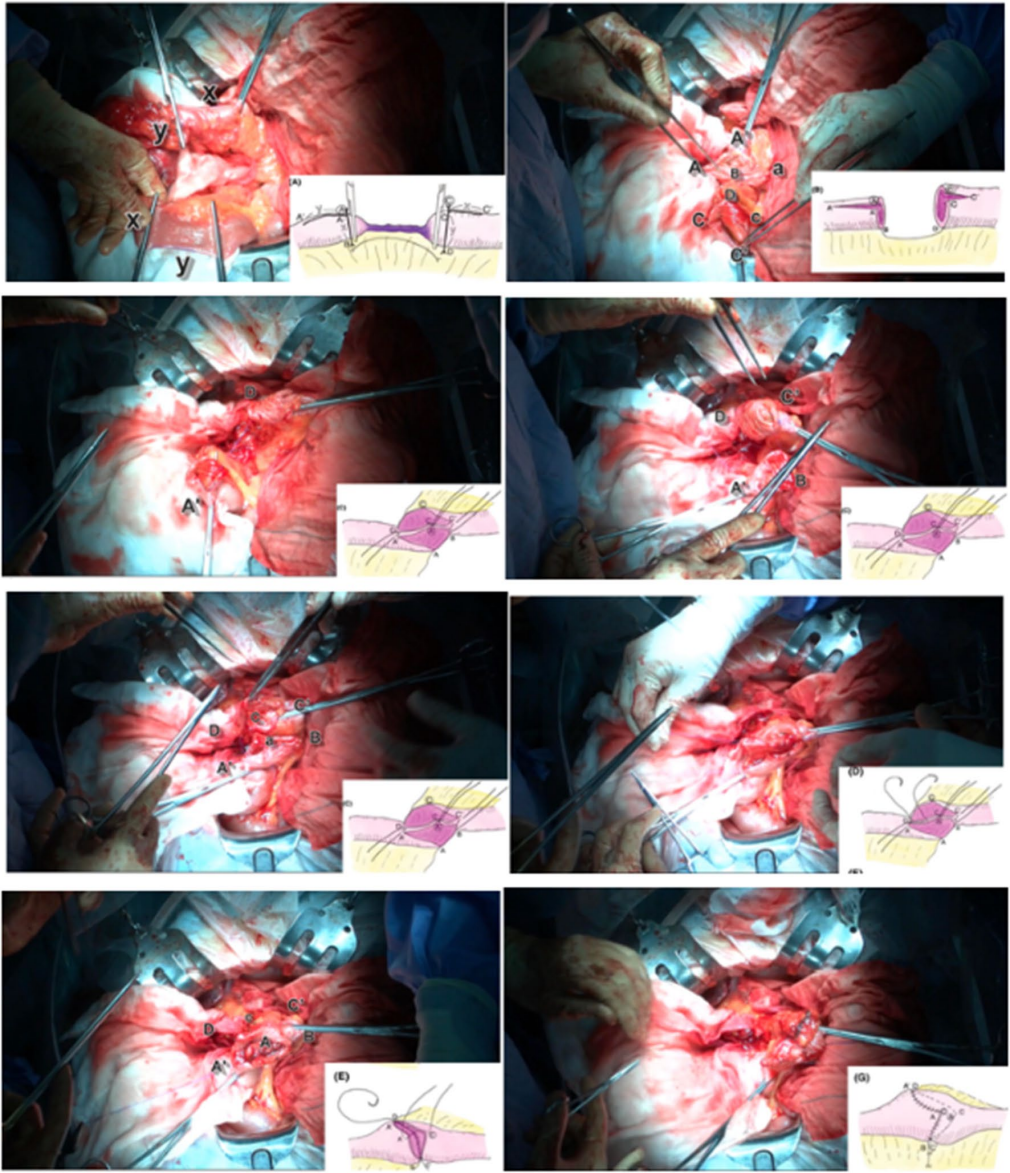
The Sasaki-W anastomosis performed in the present patient

A 30-year-old male was diagnosed with CD and treated with mesalamine and adalimumab, and underwent ileocecal resection reconstructed with the Kono-S anastomosis one month after the initial diagnosis. He was treated with infliximab without any symptoms or recurrence for 7 years at which time he presented with upper abdominal pain. Physical examination showed mild tenderness and distension in the upper abdomen. Laboratory data were unremarkable with a leukocyte count of 55.0 × 10^9^ cells/L. Computed tomography (CT) scan showed wall thickening in the ileum with proximal dilation and fluid retention (Fig. 1). Non-operative treatment with antibiotics and fasting did not improve these symptoms (Fig. 2). Since the preoperative CT scan showed > 10 cm stenosis in the ileum, endoscopic balloon dilation was not indicated. Ten days after admission, an ileocecal resection reconstructed with the Sasaki-W anastomosis was performed. At operation, a 15-cm intestinal stenosis at the site of the previous Kono-S anastomosis was found, and the transverse colon and distal small intestine reconstructed with the Sasaki-W anastomosis (Figs. 3, 4). The operating time was 150 min and estimated blood loss was 85 ml. The postoperative course was uneventful, and the patient was discharged on postoperative day 17. The patient did not have obstructive symptoms, and no signs of restenosis was observed on CT scan 1 year postoperatively.

## Discussion

Despite advances in medical therapy, approximately 40–80% of patients with CD require surgery during their lifetime due to intestinal complications such as strictures, fistulas and abscesses [1, 6]. Recurrence after surgery for CD associated intestinal stenosis is common, and most patients develop endoscopic and clinical recurrence at the site of the anastomosis. In population-based cohort studies, endoscopic recurrence has been reported in 54% of patients and clinical recurrence in 28–45% at 5 years after surgical intervention [7]. Previous studies have shown that the extent of the surgery with respect to the length of the resected segment does not alter the rate of recurrence [8]. This raises the clinical question whether the type of surgical technique influences recurrence rates or not. Stapled side-to-side anastomoses and hand-sewn end-to-end anastomoses are thought to be the conventional anastomosis commonly used to restore gastrointestinal tract continuity following intestinal resection in CD associated surgery [9]. Previous meta-analyses have supported stapled side-to-side anastomoses over hand-sewn end-to-end anastomoses resulting in an overall lower incidence of postoperative complications including anastomotic leakage and restenosis [10]. Therefore, the current consensus supports the concept that a wide lumen configuration with a side-to-side anastomosis is standard surgical technique in these patients [8].

In 2003, Kono et al. established a new technique, which uses an antimesenteric side-to-side suture technique intended to prevent anastomotic stenosis in patients with recurrent CD [4]. One of the advantages of the Kono-S anastomosis is the creation of a supporting column located immediately behind the posterior wall of the anastomosis, which prevents flow-limiting alteration of the fecal stream and maintains the orientation and a large luminal diameter at the anastomosis. This prevents distortion and stenosis associated with recurrent disease at the anastomotic site, especially on the mesenteric side. Kono et al. first reported the outcomes of this novel technique in a comparison study of 69 patients who underwent the Kono-S anastomosis and 73 patients who underwent conventional anastomoses [4]. Surgical recurrence was significantly less in patients who had the Kono-S anastomosis compared to conventional anastomoses at 5 years postoperatively (0 vs 15%, *p* = 0.001). A recent randomized controlled trial demonstrated that endoscopic and clinical recurrence rates were significantly lower after the Kono-S anastomosis compared to conventional stapled side-to-side anastomoses [11]. However, they also reported no significant differences in patients who underwent the Kono-S anastomosis and those who had conventional anastomoses in terms of postoperative surgical recurrence after 24 months (0% vs 4.6%, *p* = 0.3).

While the Kono-S anastomosis was being widely used and evaluated, and the cumulative recurrence-free rate was reported as 98.6% at 10 years after surgery for 144 CD patients [12]. Sasaki et al. reported a new type of hand-sewn anastomosis (Sasaki-W anastomosis) for patients with CD associated stenosis in 2021 [5]. The Sasaki-W anastomosis is an antimesenteric cutback end-to-end isoperistaltic anastomosis that avoids anastomosis with the site of the mesenteric attachment by rotating the bowel 180 degrees preserving peripheral circulation of the intestine (Fig. 3). Some advantages of the Sasaki-W anastomosis include that the anastomotic shape is smooth and sufficiently wide, bowel caliber differences can be adjusted by the cutback procedure, and sufficient full-thickness suture at the site of a fragile bowel wall can be performed without narrowing the lumen. In comparison to the Kono-S anastomosis, the Sasaki-W technique is unique as it is a physiologic isoperistaltic anastomosis without a blind loop. In addition, the technique itself is simple, without leaving foreign materials in the bowel such as staples (Table 1). Sasaki et al. reported that after a total of 52 procedures (15 in early and 37 in late groups) there were no complications during the follow-up period (median: 107 months) except for one patient in the early group who underwent reoperation at 106 months after surgery [5]. The cumulative recurrence-free rate was 100% at 5 years, and 86% at 10 years in the early-stage group. There were no patients in the late group that required reoperation. The results show that the technique is a reasonable surgical option for patients with CD associated recurrences. Based on the advantages mentioned above, the Sasaki-W anastomosis needs a rather short segment of bowel for reconstruction compared to the Kono-S anastomosis which led us to select the Sasaki-W anastomosis for the present patient. The Kono-S anastomosis requires a minimum of 20 cm small-bowel, with 10 cm on each side of the anastomosis serving as the supporting column [12]. In contrast, the Sasaki-W anastomosis requires only 7 cm of the small bowel. Additionally, the Sasaki-W anastomosis is isoperistaltic, while the Kono-S anastomosis is vertical to the peristalsis. This difference in direction may facilitate the spontaneous bowel movement after the Sasaki-W anastomosis. This case report provides further evidence of the effectiveness of Sasaki-W anastomosis for recurrent small-bowel stricture in the shortened small-bowel following the Kono-S anastomosis. If the effectiveness of Sasaki-W anastomosis is confirmed by larger studies, it may become a first-line treatment option.

**Table 1:** Comparison of anastomotic techniques for small intestine with Crohn’s disease stenosis

	Sasaki-W anastomosis	Kono-S anastomosis	Hand-sewn end-to-end anastomosis
Postoperative restenosis	Low	Low	Fair
Postoperative perfusion	Good	Good	Fair
Residual material	None	Staples	Staples
Difficulty	Easy	Slightly complicated	Easy
Limitation	Influenced by adhesions and/or inflammation	Residual material	Perfusion insufficiency restenosis

## Conclusions

The Sasaki-W anastomosis may be a safe and feasible surgical strategy for patients with recurrent CD. The Sasaki-W anastomosis is a viable option for patients with postoperative recurrence after the Kono-S anastomosis.

## Data Availability

All data generated or analyzed during this study are included in this article. Further inquiries can be directed to the corresponding author.

## Abbreviations

CD: Crohn’s disease
CT: Computed tomography
